# Comparative Analysis of Fecal Microbiota in Grasscutter (*Thryonomys swinderianus*) and Other Herbivorous Livestock in Ghana

**DOI:** 10.3390/microorganisms8020265

**Published:** 2020-02-15

**Authors:** Kiyonori Kawasaki, Kenji Ohya, Tsutomu Omatsu, Yukie Katayama, Yasuhiro Takashima, Tsuyoshi Kinoshita, Justice Opare Odoi, Kotaro Sawai, Hideto Fukushi, Hirohito Ogawa, Miho Inoue-Murayama, Tetsuya Mizutani, Christopher Adenyo, Yoshiki Matsumoto, Boniface Kayang

**Affiliations:** 1 Faculty of Agriculture, Kagawa University, Kagawa 761-0795, Japan; 2 Faculty of Applied Biological Sciences, Gifu University, Gifu 501-1112, Japan; 3 Graduate School of Veterinary Sciences, Gifu University, Gifu 501-1112, Japan; 4 Faculty of Agriculture, Research and Education Center for Prevention of Global Infectious Diseases of Animals, Tokyo University of Agriculture and Technology, Tokyo 183-8538, Japan; 5 Center for Highly Advanced Integration of Nano and Life Sciences, Gifu University (G-CHAIN), Gifu 501-1193, Japan; 6 Dentistry and Pharmaceutical Sciences, Graduate School of Medicine, Okayama University, Okayama 700-0914, Japan; 7 Wildlife Research Center, Kyoto University, Kyoto 606-8203, Japan; 8 Wildlife Genome Collaborative Research Group, National Institute of Environmental Studies, Tsukuba 305-8506, Japan; 9 Livestock and Poultry Research Centre, College of Basic and Applied Sciences, University of Ghana, Accra P.O. Box LG 38, Ghana; 10 Department of Animal Science, College of Basic and Applied Sciences, University of Ghana, Accra P.O. Box LG 226, Ghana

**Keywords:** cattle, goat, grasscutter, rabbit, sheep, microbiome, *Prevotella*, *Treponema*

## Abstract

The grasscutter (also known as the greater cane rat; *Thryonomys swinderianus*) is a large rodent native to West Africa that is currently under domestication process for meat production. However, little is known about the physiology of this species. In the present study, aiming to provide information about gut microbiota of the grasscutter and better understand its physiology, we investigated the intestinal microbiota of grasscutters and compared it with that of other livestock (cattle, goat, rabbit, and sheep) using 16S rRNA metagenomics analysis. Similar to the other herbivorous animals, bacteria classified as Bacteroidales, Clostridiales, Ruminococcaceae, and Lachnospiraceae were abundant in the microbiome of grasscutters. However, *Prevotella* and *Treponema* bacteria, which have fiber fermentation ability, were especially abundant in grasscutters, where the relative abundance of these genera was higher than that in the other animals. The presence of these genera might confer grasscutters the ability to easily breakdown dietary fibers. Diets for grasscutters should be made from ingredients not consumed by humans to avoid competition for resources and the ability to digest fibers may allow the use of fiber-rich feed materials not used by humans. Our findings serve as reference and support future studies on changes in the gut microbiota of the grasscutter as domestication progresses in order to establish appropriate feeding methods and captivity conditions.

## 1. Introduction

The human population has grown by 30% in recent decades in Ghana [1], where food supply balance has been unstable, particularly in the northern area, causing severe malnutrition [2]. In order to solve food problems, it is essential to secure not only grain but also animal protein sources [2]. In the northern area of Ghana, the hunting of wild animals is the main source of protein [3], which has a serious impact on ecosystems and raises concern about the risk of zoonotic infections. Therefore, it is urgent to secure a sustainable protein source to replace wild animals [4], but it is difficult to breed large livestock (e.g., bovine or swine) or fish in the northern area, where climatic conditions are harsh. Moreover, conventional livestock (e.g., cattle, pig, and chicken) consumes large amounts of grains and, thus, compete with humans for grain crops [5]. The domestication of animals that can be raised on feed that are not consumed by humans stands as an attractive alternative. Currently, there is an on-going project in northwestern Ghana aiming to enhance the domestication of a large rodent native to West Africa called grasscutter (*Thryonomys swinderianus*, also known as the greater cane rat), whose meat is a delicacy for people in West Africa [6]. As part of this project, we developed DNA markers to support the genetic management of the grasscutter in Ghana [7]. Still, there is limited information on grasscutter physiology. 

Growing evidence indicates a close relationship between nutrient utilization and gut microbiome communities in various animals [8,9,10,11]. For example, herbivorous small hindgut fermenters get short-chain fatty acids from the bacterial fermentation of fiber carbohydrates in the cecum and essential amino acids from microbial proteins through cecotrophy [12,13,14,15]. Since the grasscutter is a herbivorous small hindgut fermenter, microorganisms living in its cecum are expected to play an important role in its digestive physiology. Moreover, the carbohydrate fermentation ability of microbiota in captive animals with unnatural feeding habits is inferior to that of wild animals [9]. Therefore, assessing the microbiota of grasscutters may help to determine the appropriate feed materials and composition for promoting domestication of grasscutters. Moreover, gut microbiota of livestock is being profiled all over the world, and the interplay between health condition or growth performance of livestock and their gut microbiota is gradually becoming clearer. However, grasscutters are still in the process of being domesticated, and there is no data on their gut microbiota.

Thus, in the present study, to form a better view of the grasscutter gut microbiome, we investigated the microbiota of grasscutters using 16S rRNA metagenomics analysis and compared it to that of conventional livestock animals.

## 2. Materials and Methods 

### 2.1. Animals and Sample Collection

**Figure 1:**
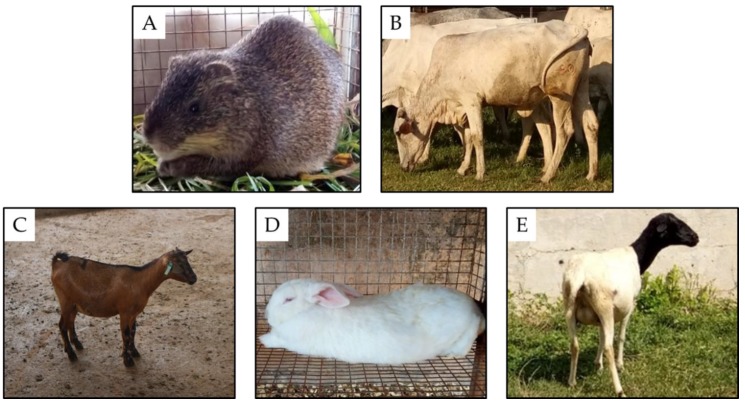
Livestock animals examined in this study: grasscutter (*Thryonomys swinderianus*; (**A**), the West African Shorthorn cattle (*Bos primigenius*; (**B**), the West African Dwarf goat (*Capra hircus*; (**C**), the New Zealand White × California White cross bred rabbit (*Oryctolagus cuniculus*; (**D**), and the Nungua Black Head sheep (*Ovis aries*; (**E**).

This research was conducted with the approval of the Gifu University animal experiment committee (Approval number: 17070; approval date: 2017.7.3) and the College of Basic and Applied Sciences Directorate (approval number was not assigned). We collected feces from 5 grasscutters and 16 livestock animals (cattle, *Bos indicus*: Sanga; goat, *Capra hircus*: the West-African Dwarf; rabbit, *Oryctolagus cuniculus*: the New Zealand white × California White; and sheep, *Ovis aries*: the Nungua Black Head; n = 4 for each species) in September 2016, 2017, and 2018 in Ghana (Figure 1). Grasscutters were purchased at the Kantamanto bushmeat market in the city of Accra, Ghana. Animals traded at the bushmeat market are hunted and brought from a wide geographical area in the coastal zone of Ghana, so their exact location is unknown. The other samples were obtained from livestock reared at the Livestock and Poultry Research Centre, University of Ghana (5°40′28″ N, 0°6′5″ W; Greater Accra; 15 km north of Accra). Feces were stored on ice, and fecal DNA extraction was conducted within 1 h after sampling.

### 2.2. Analysis of Fecal Microbiota by 16S rRNA Metagenomics Sequencing 

DNA was extracted from feces using ISOFECAL for beads beating (Nippon Gene, Tokyo, Japan) according to the manufacturer’s instructions. The V3-V4 hypervariable region of bacterial 16S rRNA genes was amplified using the universal primers 341F (5′-CCTACGGGNGGCWGCAG-3′) and 805R (5′-GACTACHVGGGTATCTAATCC-3′) [16]. The PCR reaction mixture was composed of 10 µM forward primer, 10 µM reverse primer, 2× premix Ex Taq HS (Takara Bio, Shiga, Japan), and the extracted fecal DNA template. The first PCR conditions were: initial denaturation at 94 °C for 3 min, followed by 25 cycles of 94 °C for 30 s, 55 °C for 30 s, 72 °C for 30 s, and a final extension step at 72 °C for 10 min. The second PCR conditions for index attachment were: initial denaturation at 98 °C for 30 s, followed by 8 cycles of 98 °C for 30 s, 60 °C for 30 s, 72 °C for 30 s, and a final extension step at 72 °C for 5 min. The amplicons were purified using AMPure XP beads (Beckman Coulter, Brea, CA, USA). Paired-end sequencing of all libraries was performed on an Illumina MiSeq sequencer (Illumina, San Diego, CA, USA) using a MiSeq Reagent kit v3 (600 cycles; Illumina) according to the manufacturer’s instructions. Operational taxonomic unit (OTU) identification and phylogenetic classification were performed using QIIME v2.0 [17]. The database for taxonomic assignment (identity 99%) was Greengenes (13_8 release) attached to pipeline QIIME for microbiome analysis, and all sequences not judged as chimera were extracted and used for subsequent analysis. Nucleotide sequence data reported are available in the DDBJ databases under the accession number DRA009468.

### 2.3. Statistical Analysis

Alpha diversity (Chao1 and Shannon indices) of fecal microbiota was calculated using QIIME v2.0 [17] and statistically analyzed using one-way analysis of variance (ANOVA). For β-diversity, unweighted and weighted UniFrac distances between samples were calculated using QIIME v2.0 [17], visualized by principal coordinate analysis (PCoA), and statistically analyzed using permutational multivariate analysis of variance (PERMANOVA). Figures of α and β diversity were generated using phyloseq [18]. The abundance of each bacterial genus in fecal microbiota was statistically analyzed using Welch’s t-test in the statistical analysis of metagenomic profiles (STAMP) software [19].

## 3. Results

### 3.1. Relative Abundance of Fecal Microbiota

Sequencing resulted in the identification of 37,420 OTUs among 11,741,207 (lowest 296,955; highest 1,676,171) high quality sequences. This generated a list of the ten most abundant bacterial groups (classified at the lowest possible taxonomic level) in fecal samples from grasscutter and conventional livestock animals (Table 1). Although their relative abundance differed between samples, the major microbiota groups identified in fecal samples of the animals were Bacteroidales, Clostridiales, Lachnospiraceae, and Ruminococcaceae (Table 1).

**Figure 2:**
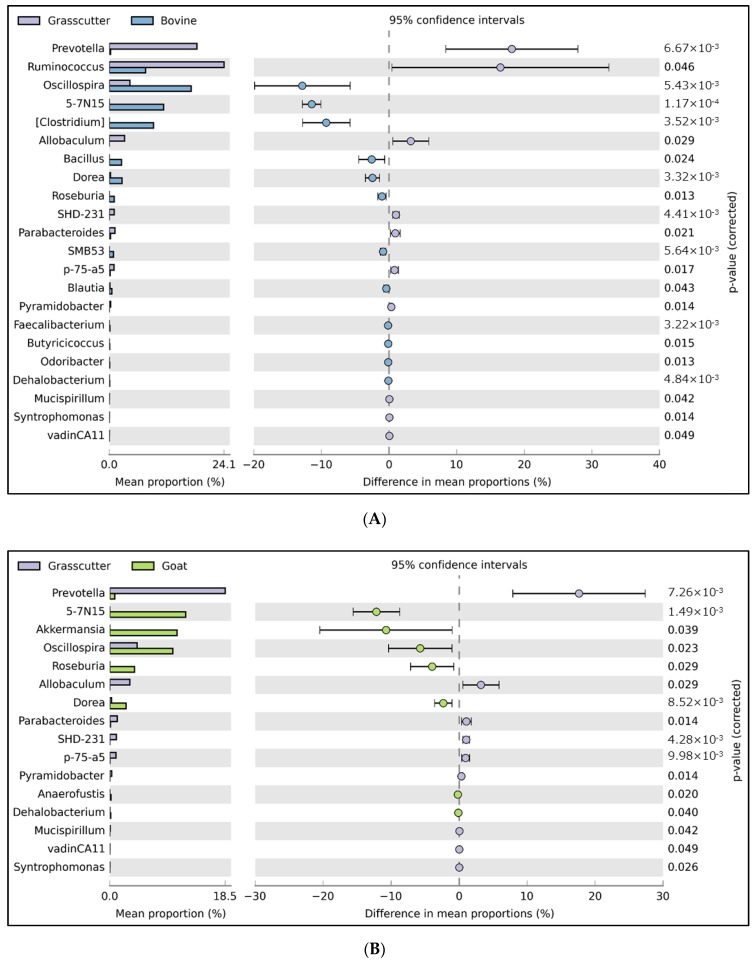

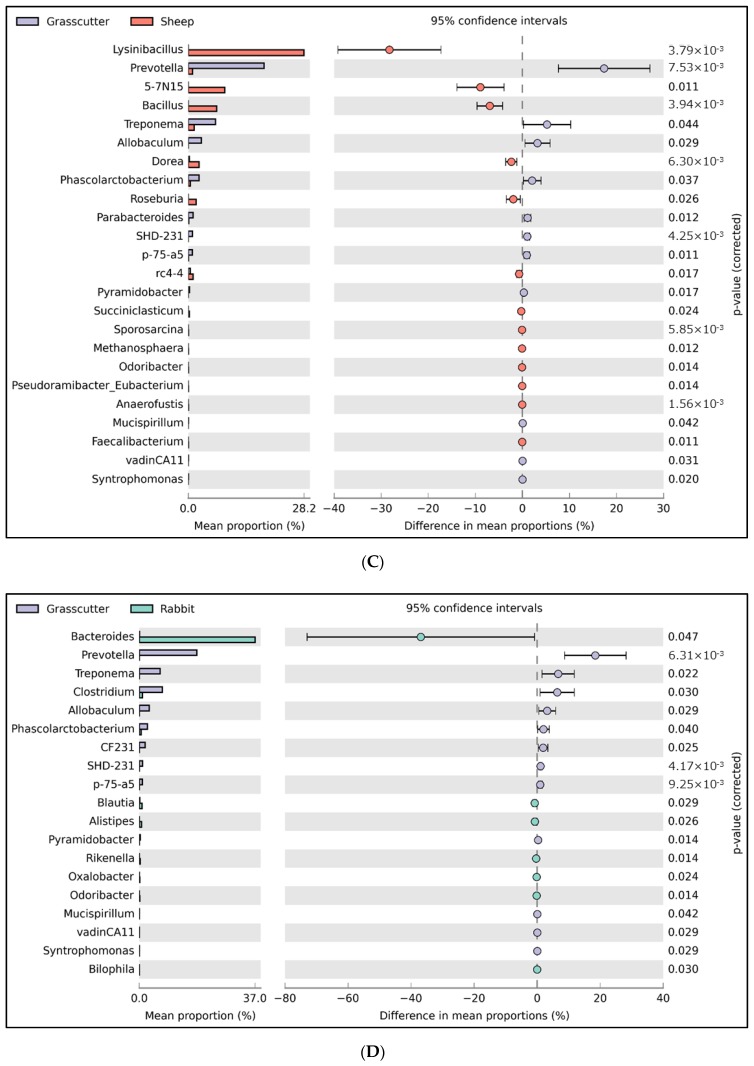
Comparative analyses of the taxonomic composition of the microbial communities at the genus level in fecal samples from grasscutter and conventional livestock. The mean proportion of representative genera that differed significantly between groups are shown as bars on the left. The differences in mean proportion of each genus, the 95% confidence intervals, and corrected P values, as calculated by statistical analysis of metagenomic profiles (STAMP) software, are shown on the right. (**A**) Grasscutter (*Thryonomys swinderianus*) versus cattle (*Bos primigenius*). (**B**) Grasscutter versus goat (*Capra hircus*). (**C**) Grasscutter versus sheep (*Ovis aries*). (**D**) Grasscutter versus rabbit (*Oryctolagus cuniculus*).

At the genus level, the mean proportion of 22, 16, 24, and 19 genera were significantly different between grasscutter and cattle, goats, sheep, and rabbits, respectively. In particular, the abundance of *Prevotella* was significantly higher in grasscutters than in the other animals (*P* < 0.05, Figure 2).

### 3.2. Analysis of Microbial Diversity for between Animals

**Figure 3:**
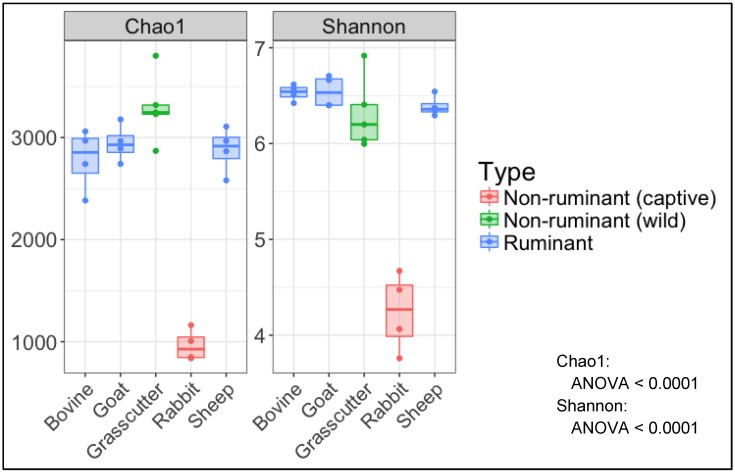
Alpha diversity indices (Chao1 and Shannon) of microbial communities in fecal samples from grasscutters (*n* = 5) and other livestock (*n* = 4).

When α-diversity (Chao 1 index: richness, Shannon index: evenness) was compared among the animals, both indices were significantly lower in rabbits than those in the other animals (*P* < 0.05); however, no difference between grasscutters and ruminant livestock was observed for any α-diversity index (Figure 3). 

**Figure 4:**
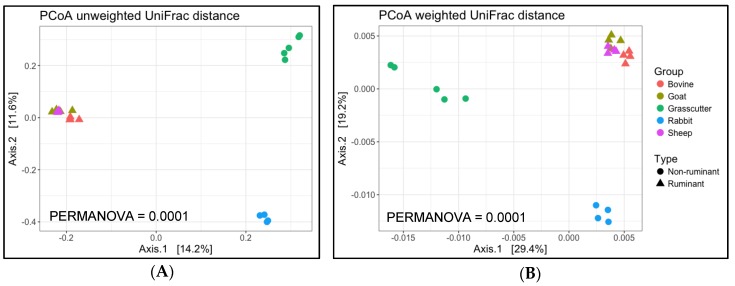
Beta diversity of microbial communities in fecal samples from grasscutter and conventional livestock. (**A**) Unweighted and (**B**) weighted UniFrac distance principal coordinate analysis (PCoA) plots of β-diversity measures of the microbiota communities in grasscutter (*n* = 5) and other livestock (*n* = 4).

Regarding β-diversity based on unweighted and weighted UniFrac distance, ruminants were closely clustered in the PCoA plots of the first two axes (axes 1 and 2; Figure 4). Grasscutters data were located away from those of rabbits, which are also small hindgut fermenters. In unweighted UniFrac distance, grasscutters and rabbits were clustered within the same range in the first axis (axis 1) of the PCoA plot (Figure 4A). Contrastingly, in weighted UniFrac distance, rabbits and ruminants (not grasscutters) were closely clustered in the first axis (axis 1) of the PCoA plot (Figure 4B). Permutational multivariate analysis of variance indicated that the β-diversity of fecal microbiota in grasscutters was significantly different from that in other animals (PERMANOVA *P* < 0.05; Figure 4). 

## 4. Discussion

In this study, aiming to establish an appropriate feeding method for domestication of the grasscutter, we investigated its fecal microbiota and compared it with those from other herbivorous livestock in Ghana. Similar to the other animals, the relative abundance of Bacteroidales, Clostridiales, Ruminococcaceae, and Lachnospiraceae—which are the major bacteria groups in herbivores [20,21,22,23]—was high in the fecal microbiota of grasscutters. Moreover, two bacteria genera were especially abundant in the fecal microbiome of grasscutters, *Prevotella* and *Treponema*, whose levels were higher in grasscutters than those in any other animal. Some species of *Prevotella* and *Treponema* have fiber fermentation ability; *Prevotella* species make acetic acid from lignocellulose [24], and *Treponema* species are found in the intestine of termites, where they play an important role in cellulose fermentation [11,25]. The presence of such a distinctive microbiome in grasscutters, including bacteria that can breakdown dietary fibers, may confer grasscutters higher fiber digestibility than that of other herbivores. From the viewpoint of domestication, an optimal diet for grasscutters should be based on resources that are not consumed by humans, so that there is no competition. If the expected high ability to digest fiber is confirmed, grasscutters could be fed a fiber-rich diet and, thus, not compete with humans.

Comparing the α-diversity of fecal microbiota among the animals, only that of rabbits showed a low value. In rabbits, the number of OTUs in gut microbiota varies greatly [22,26,27,28]. Accordingly, in the present study, more OTUs were observed in the gut microbiome of other livestock than in that of the rabbits. Rabbits have the wash-back type of colonic separation mechanism (CSM), which is a special gastrointestinal mechanism that allows small food particles to flow into the cecum [12,13,29,30]. Since the grasscutter is also a hindgut fermenter, one may speculate that they may also have a CSM. However, unlike rabbits, grasscutters may have another kind of CSM present in rodents, the mucus-trap type of CSM. The mucus-trap mechanism allows the flow of food particles that are larger than those transported by the wash-back type into the cecum [30]. It is suggested that the wash-back CSM seals the rabbit’s cecum for microbiota fermentation [30,31,32]. In other words, rabbits might be able to selectively store microbiota, whereas other animals do not have such ability. This might lead to an increase in the abundance of dominant species in the cecal microbiome of rabbits, which may be the reason why the evenness of microbiota in rabbits was low when comparing its α-diversity with that of other animals.

In the case of the fecal microbiota β-diversity, grasscutters and rabbits were clearly separated from ruminants in the PCoA plot. Domesticated rabbits have no *Treponema* in their gut, unlike hares (*Lepus* spp.) [22,33,34]. Grasscutters and rabbits are both small hindgut fermenters, but they were plotted separately from each other in the PCoA analysis. Natural rabbit diets are composed of forbs, whereas grasscutters prefer the stem portions of grasses and other plants, which could explain the diversity difference between these species. The evident separation of grasscutters from the conventional livestock animals at both UniFrac analyses may be caused by differences in feeding habits and environment. This is supported by the observation that livestock fed with the same feed under the same husbandry conditions are plotted closely to each other. For example, ruminants and hindgut fermenters are plotted away from each other in microbiota β-diversity analyses, and ruminant living in the same environment are clustered together [35]. Therefore, the grasscutters used in this study were likely obtained from the same region. Besides, it is expected that the grasscutters microbial community structure changes, which affects how it relates to that in other livestock [20], as the domestication progresses.

In captivity, the grasscutter has been fed mainly with elephant grass (*Pennisetum purpureum*) or cassava (*Manihot esculenta*) [36,37]. In addition, farmers in the northern area of Ghana are currently feeding grasscutters elephant grass, guinea grass (*Megathyrsus maximus*), or agricultural residues of maize from the surrounding area. In African countries, wild grasscutters cause damage to corn, wheat, and grass crops [38,39]. In other words, the feed currently given by farmers is be close to what wild individuals would eat. However, considering that weight gain is one of the most important parameters in meat production, the development of feed formulations (e.g., with low fiber content) for weight gain is expected. Such an artificial diet may affect the gut microbiota of the grasscutter. Indeed, in other animals, the fecal microbiota of wild and domesticated individuals within the same species are different, and their fecal microbiota is affected by feed differences [20]. Moreover, low-fiber diets or high-protein diets can alter the diversity of gut microbiota and may cause adverse effects, such as diarrhea and reduced fertility due to obesity [40,41]. Thus, in future studies, it is crucial to assess how much feed characteristics affect the gut microbiota of domesticated grasscutters. Grasscutters are in the process of domestication, and gut microbiota of non-wild grasscutters should be analyze in the future.

The data from this study will be useful for future domestication of grasscutters, especially in terms of the relationship between feed and gut microbiota. The eventual changes in the gut microbiota of the grasscutter as domestication progresses warrant further research to support the establishment of appropriate feeding methods.

## Figures and Tables

**Table 1:** The ten most abundant microbial taxonomic groups (relative abundance, %) in fecal samples from ruminant and non-ruminant herbivorous livestock in Ghana.

	Non-ruminant				Ruminant					
	Grasscutter		Rabbit		Cattle		Goat		Sheep	
No.	Taxonomy ^1^	(%)	Taxonomy	(%)	Taxonomy	(%)	Taxonomy	(%)	Taxonomy	(%)
1	Bacteroidales	11.59	*Bacteroides*	20.89	Ruminococcaceae	28.27	Ruminococcaceae	31.29	Ruminococcaceae	25.14
2	Ruminococcaceae	10.44	Clostridiales	16.62	Clostridiales	9.79	Clostridiales	11.12	Clostridiales	9.85
3	Clostridiales	9.79	Lachnospiraceae	10.41	Bacteroidales	7.64	Bacteroidales	7.75	*Lysinibacillus*	8.74
4	*Ruminococcus*	9.15	*Ruminococcus*	7.71	*Oscillospira*	4.69	Lachnospiraceae	4.97	Bacteroidales	7.21
5	Lachnospiraceae	6.71	*Anaeroplasma*	6.29	Lachnospiraceae	4.10	Bacillales	4.16	Lachnospiraceae	4.19
6	*Prevotella*	6.66	*Akkermansia*	5.32	*5-7N15*	3.17	Christensenellaceae	3.64	Rikenellaceae	3.41
7	*Fibrobacter*	3.56	*Oscillospira*	4.73	*Clostridium*	2.73	*Lysinibacillus*	3.50	Bacillales	3.20
8	RF16	3.24	Rikenellaceae	3.84	*[Clostridium]*	2.55	*5-7N15*	3.50	*Ruminococcus*	3.07
9	*Treponema*	2.35	*Bacillus*	3.00	Rikenellaceae	2.43	*Akkermansia*	3.01	*5-7N15*	2.75
10	S24-7	1.99	Ruminococcaceae	2.99	[Mogibacteriaceae]	2.40	*Oscillospira*	2.92	Christensenellaceae	2.49
Total		65.48		81.80		67.77		75.87		70.03

^1^ Microbial classification at the lowest possible taxonomic level and their relative abundance in the fecal microbiota of grasscutters (*n* = 5) and other livestock (*n* = 4).
